# Detection of *Campylobacter* spp. in farmed sheep in Mymensingh division of Bangladesh: Prevalence, risk factors, and antimicrobial susceptibility status

**DOI:** 10.14202/vetworld.2024.245-254

**Published:** 2024-02-01

**Authors:** Md. Ashiquen Nobi, A. K. M. Ziaul Haque, M. Rafiqul Islam, Sk Shaheenur Islam, Mohammad Arif, Mahmudul Hasan Sikder, S. M. Lutful Kabir

**Affiliations:** 1 Department of Microbiology and Hygiene, Bangladesh Agricultural University, Mymensingh-2202, Bangladesh; 2 Kazi Farms Poultry Laboratory, Holding no-8/1, Floor no-A3 and A4, Padma Plaza (Opposite of Gazipur Commerce College), Chandana - Chowrasta, Gazipur-1704, Bangladesh; 3 Department of Livestock Services, Ministry of Fisheries and Livestock, Dhaka-1215, Bangladesh; 4 Department of Pharmacology, Bangladesh Agricultural University, Mymensingh-2202, Bangladesh

**Keywords:** antimicrobial resistance, *campylobacter jejuni*, identification, isolation, prevalence, risk factors, sheep

## Abstract

**Background and Aim:**

*Campylobacter* infections in sheep may be asymptomatic or cause enteritis, ileitis, infertility, and abortion. Thus, this study aimed to estimate the prevalence of *Campylobacter* spp. in farming sheep and to detect risk factors, molecular patterns, and antimicrobial susceptibility status of these pathogens.

**Materials and Methods:**

Four hundred and eight fecal samples were collected from 12 flocks in the Mymensingh and Sherpur districts. Samples were tested by both basic (culture and biochemical tests) and molecular (initially 16S rRNA and later *hipO* gene-based polymerase chain reaction). Furthermore, the antimicrobial susceptibility status of *Campylobacter jejuni* was confirmed using disk diffusion. Flock- and animal-level data were captured using semi-structured interviews with farm owners under bivariate and multivariate logistic regression analyses to confirm the risk factors for *Campylobacter*-positive status.

**Results:**

The prevalence of *C. jejuni* staining at the animal and flock levels was 8.82% (36/408) and 66.70% (8/12), respectively. The age of sheep was identified as an important risk factor. Up to 1 year of age, sheep were 3.78 times more likely to be infected with *C. jejuni* (95% confidence interval: 1.0736–13.3146, p = 0.038). Of the 36 isolates of *C. jejuni*, all were found to be fully susceptible (100%) to gentamicin and ciprofloxacin. In this study, three antimicrobial agents, oxytetracycline, azithromycin, and ceftriaxone, were fully resistant (100%). The majority of isolates were resistant to a combination of 4–6 antimicrobial agents.

**Conclusion:**

The present study highlights the predominant maintenance of zoonotic *Campylobacter* species in sheep, and their burden on human health is enormous. Therefore, environmental, animal, and human health needs to be focused under a One Health lens to mitigate the occurrence of *Campylobacter* in farm settings and to prevent further introduction to animals and humans.

## Introduction

Since the last decade, *Campylobacter* has been considered one of the main causal agents of gastrointestinal infection worldwide in both developed and developing countries [1]. *Campylobacter jejuni* and *Campylobacter coli* account for most of the reported cases of *Campylobacter* infection in humans, whereas *C. coli* has a minor contribution to the overall burden. Food-producing animals such as poultry, cattle, sheep, pigs, and pets such as dogs and cats are considered to be associated with human *Campylobacter* infection [2]. However, a large number of animals have been shown to be reservoirs of *Campylobacter*, and there is no significant evidence of infection [2, 3]. *C. jejuni* is the major cause of human campylobacteriosis [4].

In general, poultry meat is accounted the pivotal cause of infection for human *Campylobacter*. The pathogens usually do live in the gastrointestinal tract (GIT) as commensalism of poultry species, especially commercial chickens and turkeys [5]. At the present time, ruminant species such as cattle and sheep contribute to the ecology of *Campylobacter*, which has been widely demonstrated in different geographical locations [6–10]. Ruminant-related *Campylobacters* could be spread to humans through food chains such as milk and water, contaminated environments, or even direct contact with source animals [11]. Notwithstanding, a significant source of human infection worldwide is the consumption of undercooked *Campylobacter*-contaminated poultry or lack of cleaning and sanitation during raw poultry-product handling [12]. However, source confirmation studies have elucidated that ruminant *Campylobacter* is the primary source of human infection [13–15].

Classically, *Campylobacter* inhabits the GIT tract in animals; however, it can also transfer through the epithelial barrier, resulting in bacteremia by systemic infection, abortion in ruminant animals, and sporadically causing infections in humans [16, 17].

Campylobacteriosis is primarily associated with *C. jejuni*, including *Campylobacter fetus* subsp. *fetus*, is known to cause abortion and stillbirth in reproductive sheep. Transmission occurs through oral or genital contact with contaminated feces, water, or aborted fetuses [18]. Although it is a common cause of abortion in the United Kingdom, it has been confirmed in Western Australia that it is rare. Formerly recognized as vibriosis, this disease is now referred to as campylobacteriosis.

Bangladesh has 1.34 million sheep, and the number of this species has been steadily increasing since the last decade [19], which would increase the public health burden as most of the sheep keepers of Bangladesh are landless marginalized communities [20] and are less aware of their health burden.

To the best of our knowledge, there have been no reports of sheep *Campylobacter* in Bangladesh. To understand the overall burden of the zoonotic pathogen in source animals, several reports have been published on the occurrence of *Campylobacter* in poultry [21–24] and dairy cows [25, 26] in different geographical locations in Bangladesh.

Thus, this study aimed to estimate the prevalence of *Campylobacter* spp. in farming sheep and to detect risk factors, molecular patterns, and antimicrobial susceptibility status of these pathogens.

## Materials and Methods

### Ethical approval and Informed consent

The study was approved by the Animal Welfare and Experimentation Ethics Committee (AWEEC) of Bangladesh Agricultural University [AWEEC/BAU/2021(11)]. All participants (farmers) included in this study were aged ≥18 years. All respondents were informed about the aims of the research. Verbal permissions were obtained as a considerable number of participants are illiterate, as they cannot read and write. The participants had the right to withdraw or not to participate in animal sampling from his/her farm and subsequent animal-level data collection.

### Study period and location

**Figure-1:**
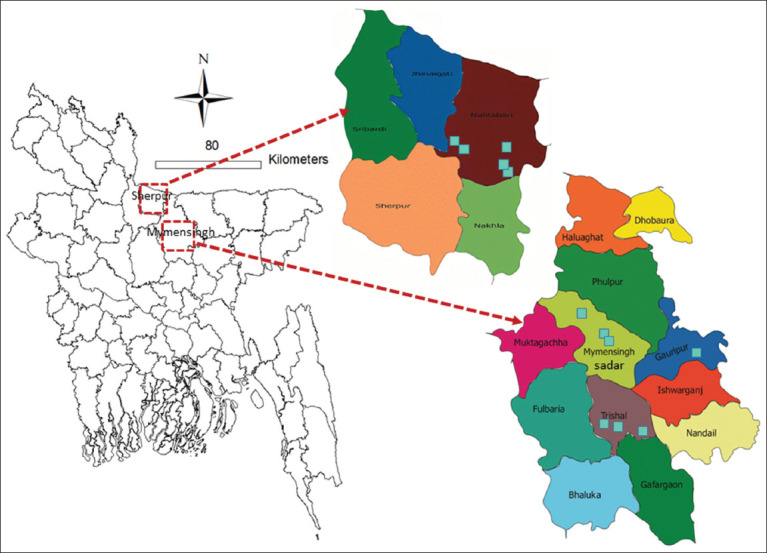
Study districts (Mymensingh and Sherpur). A total of 12 farms (five from Sherpur district and seven from Mymensingh district) were included under this study (Source: The map was generated using ArcGIS version 10.3).

The study was conducted from April to December 2021 in the Mymensigh and Sherpur districts of the Mymemsingh division of Bangladesh. Mymensingh (24°74’N, 90°40’E) and Sherpur (25° 1’ 9.8580’’ N, 90° 0’ 49.4388’’ E) are situated in the northeastern part of Bangladesh (Figure-1). These districts are located in the Jamuna Basin and are promising for profitable sheep production that could meet the meat requirement, improve livelihoods, and provide sustainable income [27].

### Selection of sheep farm and sheep

A list of sheep farms was collected from the respective upazila livestock offices located in each district. Sheep flocks with a flock size of ≥15 sheep were randomly selected.

### Sample size and sampling method

The sample size used in this study was calculated using the following equation [28].


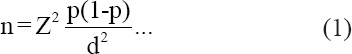


Where, n represents the requisite sample size, Z^2^ is the Z-score at a 95% confidence interval (CI) of 1.96, p is the anticipated prevalence of *Campylobacter* likely at the animal-level (53.3% = 0.53) [25], and d is the desired absolute precession (5% = 0.05); therefore, a sample size of 385 was obtained. However, we included 408 sheep from 12 farms from four upazilas (subdistricts) of two districts for animal-level sampling.

### Data collection

A data collection team consisting of a veterinarian and one veterinary field staff collected data and samples from each flock. A semi-structured questionnaire was developed and used to collect data from farmers through semi-structured interviews. The same team collected samples from each sheep flock. A questionnaire template containing determinants related to (i) flock-level characteristics (15 questions) and (ii) animal-level characteristics (nine questions). Questionnaire responses were recorded in hard copies and then stored in Excel data sheets for descriptive and inferential statistical analyses.

### Sample collection from the animals

A single fecal sample was collected from each sheep and 408 samples were collected from 12 flocks in total. Flock-level positivity status was established on the face sample evaluation status (either positive or negative). Aseptic measures were followed during sample collection. For each animal, 1–5 mL or g of swab material of feces was sampled. Each collected sample (swab material) was placed in an Eppendorf tube containing normal saline and a unique identification number was given. The sample was transported through an ice box to the designated laboratory of the Department of Microbiology and Hygiene, BAU, Mymensingh, for evaluation, maintaining a cool chain at 4°C–6°C.

### Laboratory evaluation

#### Culture and biochemical tests

Samples were independently assessed using a cellulose filter with a porosity of 0.45 m (Biotech, Göttingen, Germany) and filtration. This filter paper size is excellent for retaining 90% of the cells [29] and high flow rates could enable optimal colony growth. *Campylobacter* culture was accomplished in selective media using the standard method described by Bolton *et al*. [29] with minor adjustments. Briefly, 100 μL of each collected sample was blow-out on filters that were kept onto the surface of blood agar base no. 2 (HiMedia, Mumbai, India) (supplemented with 5% sheep blood) with Skirrow supplement for both *C. jejuni* and *C. coli* (HiMedia) and maintained at 42°C for 30 min. After removing the filter from Skirrow and/or growth-supplemented blood agar, the plates were incubated at 37°C for approximately 48 h in a microaerophilic environment using AnaeroPouch®-MicroAero (Mitsubishi Gas Chemical Co., Inc., Tokyo, Japan) for enrichment.

After 48 h, the incubated media were evaluated for the growth of bacteria. Grey, flat, and intermittently spreading colonies were observed on the surface of the media. The colony was stained using Gram’s staining method and observed under a light microscope to confirm the presence of Gram-negative curve structures. A few selected colonies from the agar media were then subcultured on the supplemented Blood agar base no. 2. Based on growth characteristics, different biochemical tests were performed according to standard methods to confirm *Campylobacter* spp. [30–32]. *C. jejuni* tested positive in biochemical tests, such as catalase, oxidase, hippurate hydrolysis, nitrate reduction, indoxyl acetate, and 1% glycerin. However, the triple sugar iron test showed a negative result.

#### Molecular detection through polymerase chain reaction (PCR)

Culture-positive isolates were provisionally confirmed as *Campylobacter* spp. by biochemical tests and PCR assays, respectively. The boiling method was used to extract DNA from a pure culture of *Campylobacter* spp. [33].

#### Detection of Campylobacter spp.

In this procedure, the genus *Campylobacter* was confirmed through 16S rRNA gene amplification using oligonucleotide primers in accordance with the standard procedure [34]. For the detection of *Campylobacter* spp., primers 16S9F-16S1540R and sequences (5´-3´): GAGTTTGATCCTGGCTC/AAGGAGGTGATCCAGCC with an amplicon size of 1530 bp were used. In this assay, the PCR conditions for 30 cycles were as follows: (a) Denaturation at 94°C for 30 s, (b) annealing at 47°C for 30 s, and (c) extension at 72°C for 90 s [34].

#### Confirmation of C. jejuni

In the present study, *C. jejuni* was identified using a molecular-based assay after confirmation of *Campylobacter* spp., hippuricase (*hipO)* gene-based PCR was performed using all isolates to discriminate *C. jejuni* according to a standard protocol [35]. For the detection of *C*. *jejuni* primers HIP400F–HIP1134R, sequences (5´-3´): GAAGAGGGTTTGGGTGGTG/AGCTAGCTTCGCATAATAACTTG with an amplicon size of 735 bp were utilized. In this assay, the PCR conditions for 30 cycles were as follows: (a) denaturation at 94°C for 30 s, (b) annealing at 55°C for 30 s, and (c) extension at 72°C for 45 s.

DNA templates of *C. jejuni* ATCC 33560, *C. coli* ATCC 33559, and *C. fetus* ATCC 27374 strains were used as positive controls in all PCR assays. *Escherichia coli* ATCC 25922 was used as a negative control. PCR products were visualized using gel electrophoresis (1.5% agarose, Invitrogen, Carlsbad, CA, USA). After staining with ethidium bromide (0.5 μg/mL) and recoloring with distilled water for 10 min, further gel images were captured using an ultraviolet transilluminator (Biometra, Göttingen, Germany).

#### Antimicrobial susceptibility testing

The antimicrobial susceptibility pattern of isolated strains of *C. jujuni* was evaluated using the disk diffusion method [36] with eight commonly used antimicrobial agents, namely, amoxicillin (AMX) (30 μg), oxytetracycline (OTE) (30 μg), gentamicin (GEN) (10 μg), streptomycin (10 μg), erythromycin (ERY) (30 μg), azithromycin (AZM) (30 μg), and ciprofloxacin (CIP) (5 μg) and ceftriaxone (CRO) (30 μg) (HiMedia). As per the protocol of the Clinical and Laboratory Standard Institute [37], we compared the growth zone of inhibition with the zone diameter recommended as resistant (R), intermediate resistant (I), or susceptible (S) to the assigned antimicrobial agents. The *E. coli* strain ATCC 25922 was employed in this evaluation as a quality measure organism. The results of antimicrobial susceptibility patterns were accomplished by performing at least double the disk diffusion method.

### Statistical analysis

Data obtained through the semi-structured interviews (SSI) and laboratory interpretation was recorded in a Microsoft Excel 2017 sheet (Microsoft Office, Washington, USA). Data quality was checked for completeness and uniformity and exported to STATA 13 (USA, StataCrop, 4905, Lakeway Drive, College Station, Texas, 77845, USA) for analysis.

Data on flock and animal risk factors were summarized using descriptive statistics. In this evaluation, frequency and proportion were estimated for categorical variables. In the logistic regression analysis, all continuous determinants, such as sheep age and body weight, were classified according to the analysis prerequisite. The herd level data were not suitable for logistic regression analysis due to the small size. Therefore, a Chi-square test was performed to evaluate the association of flock-level positive status with the risk factors. Therefore, p ≤ 0.05 was used to determine statistical significance.

Both bivariate and multivariate logistic analyses were performed to identify determinants associated with the occurrence of *C. jejuni* at the animal-level. p = 0.2 was considered as a screening standard for the inclusion of variables in the multivariable regression analysis in this study. p < 0.05 was considered to indicate statistical significance in this analysis. Determinants were entered into the multivariate model using the forward stepwise regression method.

## Results

### Descriptive epidemiology

A total of 408 fecal samples from 12 flocks of two districts were collected for bacteriological evaluations (one sample per animal). Of the surveyed farms, 58.3% (n = 7) from Mymensingh district and 41.7% (n = 5) from Sherpur district were included in this study. The majority of farms (67.7%, n = 8) were 5 years old, and 75% (n = 9) farms fed their sheep through a conventional feeding system (free-ranging/scavenging). All farms (n = 12) practiced peste des petits ruminants vaccination for immunization and did not raise any other animals (Table-1).

**Table-1:** Characteristics of flock composition, management practices, and flock level prevalence (n = 12 sheep farm).

Variables	Positive	Prevalence (%)	95% CI	p-value
Number of flocks/farms (n = 12)	8	66.70	34.9–90.1	-
District				
Mymensingh (n = 7)	5	71.40	29.0–96.3	0.67
Sherpur (n = 5)	3	60.00	14.7–94.7	
Age of the farm				
>5 years (n = 8)	7	87.50	47.3–99.7	0.0303
1–5 years (n = 4)	1	25.00	0.6–80.6	
Feeding practice				
Conventional (free ranging) (n = 9)	7	77.80	40.0–97.2	0.1572
Stall feeding (n = 3)	1	33.30	0.8–90.6	
Use vaccine (Peste des petits ruminants)				
Yes (n = 12)	8	66.70	34.9–90.1	-
Raise other animals				
No (n = 12)	8	66.70	34.9–90.1	-
Sheep shed type				
Newly build (within a year) (n = 4)	3	75.00	19.4–99.4	0.6650
Old (more than 1 year) (n = 8)	5	62.50	24.5–91.5	
Floor condition				
Dry (n = 5)	3	60.00	14.7–94.7	0.6788
Wet (n = 7)	5	71.40	29.0–96.3	
Access of sunlight				
No (n = 7)	5	71.40	29.0–96.3	0.6788
Yes (n = 5)	3	60.00	14.7–94.7	
Air ventilation				
Bad (n = 7)	5	71.40	29.0–96.3	0.6788
Good (n = 5)	3	60.00	14.7–94.7	
Veterinary health-care facilities				
No (n = 7)	4	57.10	18.4–90.1	0.4076
Yes (n = 5)	4	80.00	28.4–99.5	
Deworming				
No (n = 5)	2	40.00	5.3–85.3	0.0976
Yes (n = 7)	6	85.70	42.1–99.6	
Cleaning and disinfection				
Good practices (n = 5)	3	60.00	14.67–94.72	0.6788
Poor/no practice (n = 7)	5	71.40	29.04–96.33	
Faces use				
Aquaculture (n = 3)	1	33.30	0.8–90.6	0.1535
Fertilizer (n = 9)	7	77.80	40.0–97.2	
History of diarrhea				
Yes (n = 8)	7	87.50	47.3–99.7	0.0002
No (n = 4)	1	25.00	0.6–80.6	

CI=Confidence interval

### Prevalence and risk factors of flock-level

In this study, the flock-level prevalence was 66.70% (95% CI: 34.9–90.1). Among the districts, Mymensingh has a higher prevalence (71.40%) than Sherpur district. However, it was found to be non-significant (Table-1). Older farms (>5 years) and those with a history of diarrhea demonstrated high levels of contamination with *C. jejuni* at farm-level (p < 0.05). Other factors of flock levels were found to be non-significant (Table-1).

### Animal-level prevalence and risk factors

**Figure-2:**
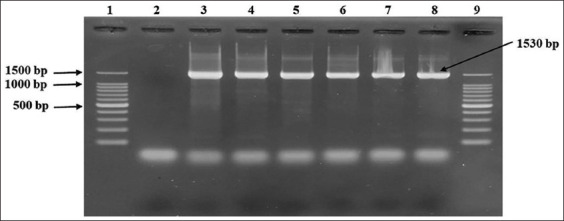
Detection of *Campylobacter* spp. by 16S rRNA gene-based polymerase chain reaction. Here, 1 and 9: 100 bp DNA ladder (Takara, Japan); Lane 4–8: Representative *Campylobacter* isolates of sheep origin; 3: Positive control (*Campylobacter jejuni* ATCC 33560); 2= Negative control.

**Figure-3:**
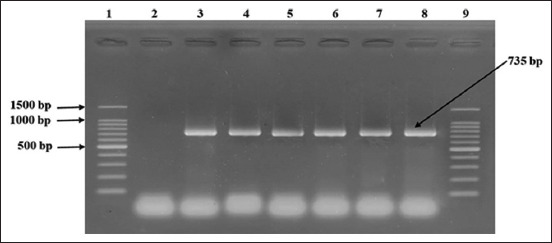
Detection of *Campylobacter jejuni* by *hipO* gene-based polymerase chain reaction. Here, 1 and 9: 100 bp DNA ladder (Takara, Japan); 2: Negative control; 3: Positive control (*C. jejuni* ATCC 33560); Lane 4–8: Representative *C. jejuni* isolates of sheep origin.

Of the 408 fecal samples, 36 were found to be positive through culture, biochemical tests, and molecular-based assays (Figures-2 and 3); thus, an animal-level prevalence of 8.8% (95% CI: 6.3%–12.0%) was confirmed (Table-2).

**Table-2:** Bivariable analysis of animal-level risk factors analysis (n = 408).

Risk factor	Category	Positive (%)	ORR	(95% CI)	p-value
Sex	Male (n = 172)	20 (11.6)	0.55	0.27–1.10	0.05
	Female (236)	16 (6.8)	Ref		
Breed	Local (270)	26 (9.6)	Ref		
	Crossbred/Garole (n = 138)	10 (7.2)	0.733	0.34–1.56	0.21
Age of the sheep	Up to 1 year (n = 36)	33 (91.7)	3.88	1.16–12.93	0.006
	>1 year (n = 372)	3 (0.81)	Ref		
Source	Bought (182)	17 (9.3)	1.122	0.56–2.22	0.37
	Farm (226)	19 (8.4)	Ref		
Body weight	≤10 kg (n = 300)	29 (9.7)	1.54	0.65–3.63	0.163
	>10 kg (n = 108)	7 (6.5)	Ref		
Pregnancy status (n = 232)	Pregnant (n = 69)	3 (4.4)	0.48	0.13–1.74	0.25
	Non-pregnant (n = 163)	14 (8.6)	Ref		
Parity (n = 68)	1–2 (n = 35)	3 (8.6%)	-	-	-
	3–5 (n = 33)	0 (0%)			
Body condition score	Bad/medium (n = 369)	35 (9.5)	3.98	0.53–29.89	0.07
	Good (n = 39)	1 (2.6)	Ref		
Season	Summer/rainy season (n = 373)	35 (9.4)	3.52	0.46–26.5	0.09
	Winter (n = 35)	1 (2.9)	Ref		

CI=Confidence interval, ORR=Objective response rate

A total of nine animal-level determinates were included in the bivariable analysis, of which two, sex and age, were found to be statistically significant (Table-2). Among the variables, four (sex, age, body weight, and body condition score) were considered as candidate variables for further multivariable model analysis (Table-3). The most important risk factor identified by this model was the age of the sheep. A 3.78-fold (95% CI: 1.0736–13.3146, p = 0.038) higher likelihood of infection with *C. jejuni* was observed in sheep up to 1 year of age (Table-3).

**Table-3:** Multivariable logistic regression analysis of *Campylobacter jejuni* infection in sheep (n = 408).

Risk factors	Adjusted odds ratio	95% C.I.	p-value
Sex			
Male	0.5379	0.2653–1.0905	0.0855
Female	Ref		
Body weight			
≤10 kg	0.7259	0.3277–1.6078	0.4298
>10 kg	Ref		
Age of the sheep			
Up to 1 year	3.7809	1.0736–13.3146	0.0384
>1 year	Ref		
Body condition score			
Bad/medium	1.5464	0.1836–13.0274	0.6885
Good	Ref		

CI=Confidence interval

### Antimicrobial profile

#### Antimicrobial susceptibility status

Of the 36 isolates of *C. jejuni*, all were found to be fully susceptible (100%) to GEN and CIP. Three antimicrobial agents, OTE, AZM, and CRO, were fully R (100%) in this study. However, some antimicrobial agents, such as AMX (11.11%, n = 4), streptomycin (5.56%, n = 2), and ERY (2.78%, n = 1), were mildly susceptible in the present study. Four antimicrobials, (AMX; 8.33%) streptomycin (S; 11.11%), (ERY; 11.11%), and AZM (AZM; 38.89%) were documented as intermediate outcomes of resistance/susceptibility (Table-4).

**Table-4:** Antimicrobial susceptibility status of *C. jejuni* isolates.

Antimicrobial agents	No. (%) of *C. jejuni* isolates		

	Resistant	Intermediate	Susceptible
Amoxicillin	29 (80.56)	3 (8.33)	4 (11.11)
Oxytetracycline	36 (100.00)	0 (0.00)	0 (0.00)
Gentamicin	0 (0.00)	0 (0.00)	36 (100.00)
Streptomycin	30 (83.33)	4 (11.11)	2 (5.56)
Erythromycin	31 (86.11)	4 (11.11)	1 (2.78)
Azithromycin	22 (61.11)	14 (38.89)	0 (0.00)
Ciprofloxacin	0 (0.00)	0 (0.00)	36 (100.00)
Ceftriaxone	36 (100.00)	0 (0.00)	0 (0.00)

*C. jejuni=Campylobacter jejuni*

### Antimicrobial resistance (AMR) status

**Figure-4:**
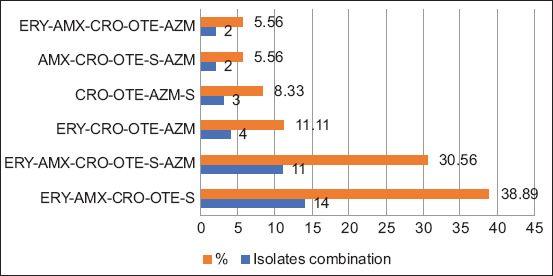
Distribution of antimicrobial resistance pattern in *Campylobacter jejuni* isolates (n = 36) from sheep. ERY=Erythromycin, AMX=Amoxicillin, CRO=Ciprofloxacin, OTE=Oxytetracycline, AZM=Azithromycin, S=Streptomycin.

In this study, four antimicrobial agents (ERY-CRO-OTE-AZM and CRO-OTE-AZM-S) were R to 11.1% (n = 4) and 8.3% (n = 3) isolates of 36 isolates through two different antimicrobial combinations. However, three different antimicrobial combinations of five antimicrobial agents (ERY-AMX-CRO-OTE-S, AMX-CRO-OTE-S-AZM, ERY-AMX-CRO-OTE-AZM) were found to be R to 38.9% (n = 14), 5.6% (n = 2), and 30.6% (n = 11) isolates, respectively. Alternatively, 30.6% (n = 11) isolates were R to six antimicrobial agents (ERY-AMX-CRO-OTE-S-AZM) (Figure-4).

## Discussion

This is a maiden study in Bangladesh that confirms the distribution of *C. jejuni* in semi-scavenging sheep in the Mymensingh division of Bangladesh and confirms the prevalence, molecular pattern, antimicrobial susceptibility status, and risk factors of *Campylobacter* infection.

The estimated farm-level occurrence of *C. jejuni* was 66.70% (95% CI: 34.9–91.1), with an estimated animal/sample-level prevalence of 8.8% (95% CI: 6.3%–12.0%). Younger animals (1 year old) were more likely to be associated with *C. jejuni* infection in semi-scavenging sheep from the farms studied. The results of this study have highlighted the overall risk of zoonotic pathogens in the source animal species. In addition, the study suggests plausible risk reduction options such as cleaning and sanitation, including hygienic practices at the farm-level, which could impede the pathogen transmission cycle at the animal-human interface.

This study documented an animal-level prevalence of 8.8% (36/408) through fecal sample evaluation. There are no published reports on *Campylobacter* occurrence in Bangladesh; therefore, we were unable to compare this data with previous records in Bangladesh. A study in Bangladesh confirmed that the overall prevalence of *Campylobacter* spp. and *C. jejuni* in farmed dairy cattle was 18% and 12.6%, respectively [25]. However, another study reported a prevalence of 25% (20/80) in different samples collected from crossbred high-yield dairy cattle [26]. However, in Bangladesh, several studies using poultry and environmental samples from live bird markets have confirmed that the prevalence rate of *Campylobacter* varies from 26.4% to 75% [21–24]. The prevalence of *Campylobacter* spp. was 9.33% in sheep in the coastal region of Odisha, India [38] and 12.5% in Kashmir, India, according to vaginal swabs and aborted material examination [39]. The findings of these studies corroborated with our study findings.

However, several studies in different geographical locations have confirmed the prevalence of *Campylobacter* in sheep, namely, 13% in Algeria [40] and 18.6% in Ghana [41]. The findings of these studies are narrowly consistent with those of our study.

In flock-level risk factor evaluation, older farms (>5 years) were more likely to be infected with *C. jejuni* (p = 0.0303). The findings of this study are consistent with those of another study conducted on dairy cattle in Bangladesh, in which >5-year-old cattle farms were found to be >10 times more risky with a *Campylobacter*-positive outcome [25]. In the case of poultry, older farms in European countries have also been reported to be *Campylobacter*-positive [42]. A history of diarrhea (p = 0.0002) was also found to be associated with the *Campylobacter*-positive status of the flock in this study.

In multivariable logistic regression analysis, four factors (sex, body weight, age of the sheep, and body condition score) were included in the animal-level risk factor evaluation: The odds of *Campylobacter*-positive status were 3.78 (95% CI: 1.0736–11.3246) times higher in sheep aged >1 year compared to sheep aged >1 year. This organism can grow in the rumen and live as a commensal organism. However, in young animals with immature rumen, a favorable condition for infection in the lower part of the GIT could be observed [11]. Thus, in this study, young animals were estimated to have a higher risk of *Campylobacter* occurrence.

The emergence of *Campylobacter*-related AMR is a persistent public health problem. Considering a vital public health risk, the World Health Organization has recorded multidrug-R (MDR) bacteria [43–45]. *C. jejuni*, *C. coli*, and *C. fetus* have been confirmed in dairy cattle, bulls, and poultry, including live bird market environmental samples in Bangladesh [21–26, 46]. However, systematic screening focusing on the antimicrobial susceptibility pattern of *Campylobacter* isolates in semi-scavenging sheep has been less considered. Thus, the data generated in this study can be used as helpful reference information.

In this study, several antimicrobial agents, such as OTE (100%), CRO (100%), streptomycin (83.33%), ERY (86.11%), AMX (80.56%), and AZM (61.11%), were documented to be R to *C. jejuni* isolates, which is an immense public health concern. The same pattern of AMR to these antibiotics has also been reported in *C. jejuni*, *C. coli*, and *C. fetus* in poultry and livestock from Bangladesh [21–26, 46]. The antimicrobial susceptibility rate of CIP and gentamycin was confirmed to be 100% for both antibiotics. The reported susceptibility rate of CIP has been confirmed in different geographical locations [47–49]. In this study, very few antimicrobial agents were documented as I/susceptible with variable proportions, such as AZM, streptomycin, ERY, and AMX in 38.89%, 11.11%, 11.11%, and 8.33%, respectively. This phenomenon has developed due to the inappropriate use of antimicrobial agents in animal production in Bangladesh [50].

A notable finding of this study was the high prevalence of MDR pathogens in sheep samples showing registrants against four to six antimicrobial agents with different combinations, such as ERY-CRO-OTE-AZM and CRO-OTE-AZM-S, AMX-CRO-OTE-AZM and AMX-CRO-OTE-S-AZM, and ERY-AMX-CRO-OTE-S-AZM. This study observed a variable distribution (5.5%–38.9%) of MDR *C. jejuni* isolates, sanitation. This distribution is sparsely consistent with other studies in poultry and livestock in Bangladesh [21, 23, 26]. In a previous report, *C. jejuni* isolates were documented to be MDR against tetracycline, ampicillin, norfloxacin, and nalidixic acid; however, the MDR status of *C. coli* was reported to be R against tetracycline, ampicillin, ERY, norfloxacin, and nalidixic acid [51].

The data obtained in this study would be helpful for the systematic assessment of zoonotic *Campylobacter* infection in sheep and its subsequent transmission in humans and the environment. The outcomes of this research will assist in making evidence-based decisions and ranking control options, such as farm cleaning, sanitation, and personal hygiene of the animal attendants.

In Bangladesh, *C. jejuni* is the primary causative agent of diarrhea in young children (25.5%) [52]. *Campylobacter* infection causes acute flaccid paralysis associated with Guillain-Barré syndrome (GBS) and has been confirmed in Bangladesh with an expected incidence of 3.25 cases/100,000 children aged 15 years [53, 54]. Many measures have been taken to minimize the burden of *Campylobacter* infection, including associated GBS threats, without considering the sources of infection in low-resource settings. Therefore, consideration should be given to the significant hazard of *Campylobacter* present in source animals such as sheep. A comprehensive understanding of the forms of release of *Campylobacter* by animals on a farm and the relationship between host animals and the environment, including pathogen genotypes, is crucial for the application of appropriate intervention approaches.

### Limitations

Identification of *Campylobacter* spp. in farmed sheep relied on fecal samples. It should be noted; however, that the survey did not collect data on the history of abortion, premature parturition, or other pathological lesions commonly associated with *Campylobacter* infection. Collecting these data would strengthen the validity of our findings. Therefore, we recommend conducting a future survey with a broader range of sample sizes to address these issues.

## Conclusion

This study highlighted the widespread presence of zoonotic *Campylobacter* in sheep, emphasizing its role in environmental contamination and posing a public health burden. To minimize the increasing burden of *Campylobacter* transmission from animals to humans, it is essential to develop innovative approaches, therapies, and interventions. To mitigate the occurrence of *Campylobacter* in farms and prevent its further transmission between animals and humans, it is essential to adopt a “one health” approach focusing on environmental, animal, and human health.

## Authors’ Contributions

MAN, AKMZH, MRI: Contributed to the field survey and data and sample collection from sheep. MAN, MA, and MRI: Performed laboratory assessment and drafted the manuscript. SSI: Performed statistical analysis of the data and reviewed the manuscript. MHS and SMLK: Supervised the study and reviewed the manuscript. All authors have read, reviewed, and approved the final manuscript.
